# Is scientific reform an unwinnable arms race?

**DOI:** 10.1371/journal.pbio.3003660

**Published:** 2026-02-11

**Authors:** Marcus R. Munafò, George Davey Smith

**Affiliations:** 1 Vice Chancellor’s Office, University of Bath, Bath, United Kingdom; 2 MRC Integrative Epidemiology Unit at the University of Bristol, Bristol, United Kingdom

## Abstract

Methodological improvements should, in theory, mean more robust evidence and inference, and more rapid advances in knowledge. However, these methods are often subsequently used in the pursuit of publication for the sake of publication, rather than in the pursuit of knowledge. Can we break this cycle?

## Teaser

Improvements to scientific methods and approaches should, in theory, mean more robust evidence and inference, and more rapid advances in knowledge. In practice, they have led to an increasing number of poor-quality studies. Can we break this cycle?

In the 1990s, Doug Altman wrote: “We need less research, better research, and research done for the right reasons” [1]. This remains a fair comment today; indeed, you could even say that we have gone backwards. As new scientific methods and approaches have been introduced, with the intention of improving research quality, they have also been used in the pursuit of publication for the sake of publication, rather than in the pursuit of knowledge. Journals are being swamped by manuscripts of increasingly minimal or even negative scholarly value, even as ever more journals are being launched to deal with a tidal wave of research. We need to act now to stem the tide.

Take our field of epidemiology as an example. At the turn of the millennium, the field was widely seen to be in crisis, with high-profile commentaries suggesting that it was reaching its limits [2] and, despite notable early successes such as identifying harms caused by smoking, was now erroneously making everyday life appear menacing [3]. This was stimulated by the many factors that were identified as being either harmful or protective in apparently high-quality epidemiological studies, which were then subsequently refuted in randomized controlled trials. The development of novel causal inference methods—particularly mendelian randomization (MR), which incorporated the burgeoning field of genetic epidemiology—was one response to this impasse [4]. Early signs were encouraging; where we were confident that we already knew the correct answer to a causal question (e.g., “Does smoking cause lung cancer?”) MR gave the right answer. It also provided important novel insights, such as challenging the notion that raising levels of HDL (“good”) cholesterol would reduce the risk of myocardial infarction [5]. As genome-wide association studies (GWAS) proliferated and summary data from these were shared, MR methodologists developed approaches that could readily make use of these data. The result was some powerful studies, but these have since been swamped by an explosion of studies that simply combine a putative exposure with a putative outcome (often seemingly at random), apply the method unthinkingly (and increasingly use AI tools to write the paper), and then submit the resulting product to a journal [6].

Making research data and other intermediate research outputs more freely available should accelerate the production of knowledge and improve quality through error detection (both of which are demonstrably at least partly true). But now we are seeing readily available data assets, such as GWAS summary statistics and observational cohort study data from the UK Biobank, NHANES, and many other databases, being weaponized to generate manuscripts with minimal scholarly value and utility (or even negative utility given the burden they place on the system) [7]. In many ways, the story of MR recapitulated what had happened previously with meta-analysis [8]. The aggregation of existing data from combinable randomized trials led to many important clinical and public health advances, and to less research waste and better science going forward. However, the combination of using data that could be extracted from existing publications with relatively simple statistical analyses led to an explosion of publications of decreasing and then negative scientific value. Thus, despite best intentions, efforts to improve research rigor, such as by making data available, synthesizing evidence, and improving causal inference methods, have become commodified by authors and paper mills to increase the marketability of a manuscript, rather than to improve the quality of scholarship [9].

Why is change so difficult? Perhaps an under-appreciated aspect of the research ecosystem is that it is dynamic—it adapts. A new approach, introduced in the hope of improving things, becomes incorporated by the system. In the sociological/political sense, this is termed recuperation: the new approach is absorbed, neutralized, and re-incorporated into the very process it was intended to improve [10]. This has played out in the cases of meta-analysis and MR; whilst important work is still produced, it becomes swamped by publications of minimal value. Ultimately (in our view), this happens because the currency of scholarship—publication—is becoming increasingly detached from the notion that publication is meant to add to the sum of human knowledge. In other words, publication has increasingly become an end in itself. It is no longer simply a means by which we communicate knowledge, it is how we advance our careers. Mere productivity (volume of output) continues to dominate, despite efforts to reward quantity over quality (e.g., the UK Research Excellence Framework).

Thus, efforts to improve data availability, evidence synthesis, causal inference, and so on have all had positive impacts by increasing signal. But they have also, unintentionally on the part of their advocates, resulted in a corresponding increase in noise through the proliferation of publications of minimal (or negative) utility. The degree to which the impact of these efforts (at least in the context of the overall published academic literature) has been positive, neutral, or negative is context-dependent, and remains to be fully analyzed or understood. In other words, we are in a scholarly arms race in which we must run constantly simply to stand still. Unfortunately, we seem unable to run faster than those who would subvert potential advances. As funders, institutions, and researchers embrace the importance and value of open, rigorous, and reproducible research, we also see the incorporation of these by political agendas. The processes of recuperation are more agile than those of reform.

Working in population health, we believe in the value of population-level interventions (e.g., improving our scholarly communication system rather than picking off individual flawed articles). But population-level interventions often result in countermeasures from vested interests. Publishers continue to launch new journals, thereby fueling the underlying over-production problem. For some, the allure of article processing charges appears to be greater than the need to maintain a genuinely valuable version of record. The tools we have, if correctly used, can be powerful. Altman was clear that systematic review was central to reducing bias, with meta-analysis secondary to that. But most of the meta-analyses that have proliferated in recent years have focused exclusively on the meta-analysis part and omitted a rigorous systematic review. Similarly, MR is intended to be used in the context of a wider triangulation of evidence [11], yet currently rarely is. However, in keeping with the history of recuperation in the context of scientific reform, we predict a rise in paper mill products with both MR and triangulation in their title [9], given growing interest in triangulation as a further approach to strengthening causal inference in epidemiology.

What can be done to break this cycle? Perhaps the time has come to think beyond the journal article as the primary unit of scholarly communication (and currency of scholarship). It has served us reasonably well for over 400 years, but does not reflect the dynamic, team-based nature of modern science. Initiatives like Octopus, whilst still in their infancy, offer a glimpse of a new knowledge architecture that can evolve in near real time and better capture the granular contribution of individuals and teams.

Clearly, there can be no single solution that will solve the problem of a systematic focus on quantity over quality. Indeed, to think of a solution is to make a category error. The scholarly communication system—and the incentive structures we have created around this—is not a static one that simply has a flaw to be repaired. It is a dynamic (perhaps, analogically at least, Darwinian) one that needs constant tinkering [12] so that it can, in general, thrive. Evidence-informed approaches will help us to better understand what works, but we will also need to monitor how these approaches evolve over time. Then, just maybe, we can stem the tide.

## Abbreviations

GWAS: genome-wide association studies
MR: mendelian randomization
